# Endocytosis in anaerobic parasitic protists

**DOI:** 10.1590/0074-02760240058

**Published:** 2024-07-26

**Authors:** Marlene Benchimol, Wanderley de Souza

**Affiliations:** 1 Universidade Federal do Rio de Janeiro, Centro Nacional de Biologia Estrutural e Bioimagens, Rio de Janeiro, RJ, Brasil; 2 Universidade da Grande Rio, Duque de Caxias, RJ, Brasil; 3 Universidade Federal do Rio de Janeiro, Instituto de Biofísica Carlos Chagas Filho, Laboratório de Ultraestrutura Celular Hertha Meyer, Rio de Janeiro, RJ, Brasil

**Keywords:** endocytosis, protozoan, electron microscopy, Entamoeba, Trichomonas, Giardia

## Abstract

The incorporation of different molecules by eukaryotic cells occurs through endocytosis, which is critical to the cell’s survival and ability to reproduce. Although this process has been studied in greater detail in mammalian and yeast cells, several groups working with pathogenic protists have made relevant contributions. This review analysed the most relevant data on the endocytic process in anaerobic protists (*Entamoeba histolytica*, *Giardia intestinalis*, *Trichomonas vaginalis*, and *Tritrichomonas foetus*). Many protozoa can exert endocytic activity across their entire surface and do so with great intensity, as with *E. histolytica*. The available data on the endocytic pathway and the participation of PI-3 kinase, Rab, and Rho molecular complexes is reviewed from a historical perspective.

Endocytosis, a complex and vital cell process, plays a role in various physiological and pathophysiological processes. For instance, it is involved in cell nutrition, defence mechanisms, cell growth and differentiation, and immune responses.^1^ ^-^ ^10^ It triggers the internalisation of the plasma membrane and the formation of endosomes, progressing through several intracellular compartments for sorting and routing cargo, ultimately leading to lysosomal degradation. Moreover, the formation of endocytic vesicles is one of the arms of the mechanism for maintaining the area of the plasma membrane, as it compensates for the increase in the area resulting from cell secretion and renewal of the membrane molecular components.

Endocytosis is also related to inflammation processes and signalling for cell death, such as in apoptosis and autophagy.^2^ Endocytic mechanisms affect the lipids and proteins of the plasma membrane; thus, endocytosis can regulate cells and their interactions with their environments. Cell-surface components are internalised during endocytosis via pinocytosis, phagocytosis, or receptor-mediated endocytosis, including clathrin, caveolae, and non-clathrin or caveolae-mediated mechanisms.

Endocytosis includes some categories: phagocytosis and pinocytosis. Pinocytosis occurs through distinct mechanisms, which can vary concerning the composition of the coat, the size of the detached vesicles, and the fate of internalised particles: macropinocytosis, clathrin-mediated-endocytosis, caveolin-mediated endocytosis, and clathrin/caveolin-independent endocytosis.^3^ ^,^ ^6^

The most notable coat is that formed by the polymerisation of clathrin, with the participation of several other proteins. Among them, it is worth highlighting the cellular adapter protein-2 (AP2 adapter) that interacts with the receptor, arrestin, and non-filamentous actin.^7^ ^,^ ^8^ The most distinctive feature of receptor-mediated endocytic vesicles formed with a clathrin coat is their very concentrated contents, increasing internalisation efficiency. When the vesicles detach from the plasma membrane, which can occur by the action of dynamin, the clathrin coat depolymerises. The vesicles fuse with a sequence of progressively acidic compartments called endosomes. Endosome acidification results from the action of proton pumps named V-ATPases^9^ present in all endosomes, including lysosomes.

In the first compartment, the early endosome, molecules in the vesicle lumen find a pH of about 6.5, lower than that of the extracellular medium. Therefore, receptor-ligand uncoupling occurs, allowing the receptor to return to the plasma membrane in recycling vesicles while the endocytosed macromolecules follow the degradation route. The different pathways followed by the internalised molecules have justified the denomination of sorting endosomes for the early endosomes. The late endosomes are the next compartment reached by endocytic cargo, whose pH is about 6.0. Newly synthesised hydrolases originating from the Golgi complex are also found in the late endosomes. However, most still need to be mature and can start or do the degradation process very slowly. In this compartment, the remarkable formation of internal vesicles occurs, mediated by the Endosomal Sorting Complex Required for Transport (ESCRT), ubiquitin, and phosphatidyl inositol. Because of the internal vesicles, late endosomes are also named multivesicular bodies (MVBs). Because of this process, late endosomes can drive the degradation of all membrane components that reach their lumen.^10^ The multivesicular compartment is also a precursor of organelles in specialised cells, such as the lytic granules of NK lymphocytes or the melanosomes of melanocytes.^11^ ^,^ ^12^ Late endosomes can generate exosomes in many cell types when the endosome fuses directly with the plasma membrane and releases its vesicular contents to the extracellular medium.

The endocytosed molecules reach the lysosomes to finish the degradation route. In different models, there is convincing evidence that late endosomes fuse with lysosomes, forming a short-lived hybrid organelle that reconstitutes late endosomes and lysosomes. In other models, there is evidence of fusion limited to a small area, just long enough to exchange contents, in a process called “kiss and run”. However, at all stages of the endocytic pathway, it has already been demonstrated that macromolecules can be transferred by carrier vesicles (ECVs).^13^ Inside them, acid hydrolases, such as proteases, phosphatases, glycosidases, nucleases, lipases, and phospholipases, are optimally functioning at pH 4.5-5.5. Degradation products are directed to the cytosol via special transporters in the lysosome membrane.^14^ ^,^ ^15^ The lysosomal-associated membrane protein 1 (LAMP1) is a highly glycosylated lysosome membrane protein considered an organelle marker.^16^

Parasitic protists comprise many species. Some of them constitute important agents of diseases of high interest in humans and animals of economic relevance. Examples include human diseases such as malaria, toxoplasmosis, cryptosporidiosis, Leishmaniasis, Chagas disease, amebiasis, trichomoniasis, and giardiasis. Concerning animal diseases, we can mention eimeriosis, babesiosis, theileriosis, cattle trichomoniasis, and cryptobiosis, among others.

These protists can be divided into two groups concerning their interaction with hosts. A group always remains outside host cells, with the life cycle in the bloodstream and tissues of the infected animals, in cavities such as the urinary and intestinal tract. Some protists also inhabit the digestive tract of invertebrate hosts, especially in insects, where part of their life cycle takes place. Some remain free or attached to the cells of the hosts. The protists of the other group, however, developed the ability to interact with the host cells’ surface and trigger an endocytic process that leads them to penetrate (or be internalised) into the cells, where they initially live within a special endocytic vacuole, known as parasitophorous vacuole (PV), or leave this vacuole to multiply in direct contact with the host cell cytoplasm, establishing interaction processes with some structures and organelles.

Whether the parasite lives in an extracellular or intracellular environment, it must internalise the basic nutrients necessary to run all metabolic pathways that lead to synthesising small and large molecules that ultimately constitute the cell. Such uptake involves the participation of all mechanisms of incorporation of molecules to the plasma membrane, including passive diffusion, active transport through the membrane, and endocytosis.

Some properties were lost during prokaryotic evolution to eukaryotic organisms, while others were acquired. Among the latter is the ability of eukaryotic cells to incorporate macromolecules, macromolecular complexes, and even other cells through a process that involves the formation of endocytic vesicles and vacuoles. For most eukaryotic cells, including pathogenic protists, endocytosis is the basic mechanism for ingesting macromolecules that are subsequently degraded in the endosomal-lysosomal system and provide important precursors for several key metabolic pathways, including the assembly of key macromolecules such as proteins and nucleic acids. The extent of endocytic activity varies across different protists and various developmental stages of some species. A better understanding of the mechanisms used by parasitic protists to ingest macromolecules via endocytic activity is important for at least two main reasons. First, we need to know if endocytosis is a universal property of all eukaryotic cells, even those considered more primitive, following a well-defined sequence of cellular events. Anaerobic parasitic protists are of special interest since the energy required for the endocytic activity comes mainly from glycolytic activity rather than mitochondrial metabolism.

Additionally, trichomonas is of special interest since they have hydrogenosomes, a mitochondrial-related organelle that consistently produces ATP. Second, endocytosis is vital to parasite survival. Identifying key molecules involved in such a process may constitute targets for developing new chemotherapeutic agents.

**Endocytosis in *Giardia***

*Giardia* is an intestinal parasite that infects various animal hosts (birds, reptiles, and mammals). In mammals, the infection occurs by *G. intestinalis*, synonymous with *G. lamblia* and *G. duodenalis*. This parasite has a worldwide distribution, with a 2-7% prevalence in developed countries, and can reach over 30% in low - and middle - income countries, affecting mainly children. The incidence of the disease is very high in areas with inadequate sanitary conditions and poor water treatment, where the parasite is found in its cystic form. The transmission of parasites can also occur through contaminated food. Cysts have a protective wall and differentiate into trophozoites when ingested by the host, colonising the small intestine. The most frequent symptoms of giardiasis are acute or chronic diarrhoea, abdominal colic, flatulence, dehydration, nausea, vomiting, and fatigue.

*Giardia* trophozoites present few organelles, and among them, they possess a ventral disc made of microtubules and microribbons, endoplasmic reticulum, two nuclei, and the peripheral vesicles (PVs) located underneath the plasma membrane of the trophozoites (Figs 1A, 2-3). No canonical Golgi and lysosomes have been observed up to now.

**Fig. 1:**
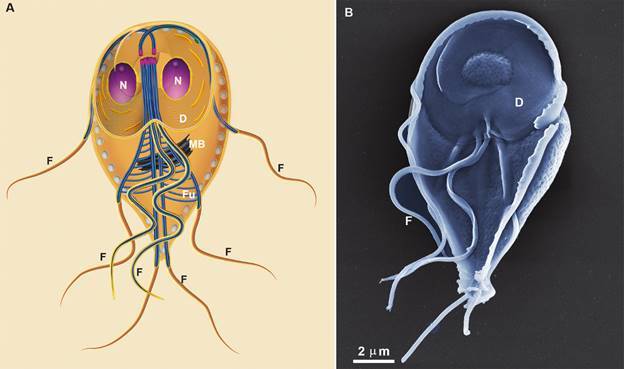
squeme (A) of *Giardia intestinalis* as seen by scanning electron microscopy (SEM) in a ventral view. D: disc; F: Flagella; Fu: funis; MB: median body; N: nucleus. Benchimol (unpublished data).

**Fig. 2:**
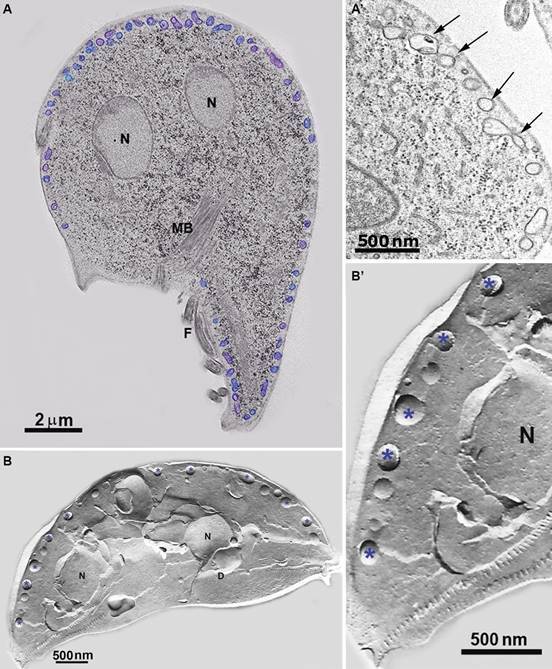
thin-section (A-A’) and freeze-fracture (B-B`) of *Giardia intestinalis*. The peripheral vesicles (PVs) (arrows and asterisks). (A) PVs are artificially coloured. (A) Arrows point PVs; in (B, B’), PVs are pointed by asterisks. Note that PVs are located just beneath the plasma membrane. N: nucleus; D: disc; MB: median body. Benchimol (unpublished data).

**Fig. 3:**
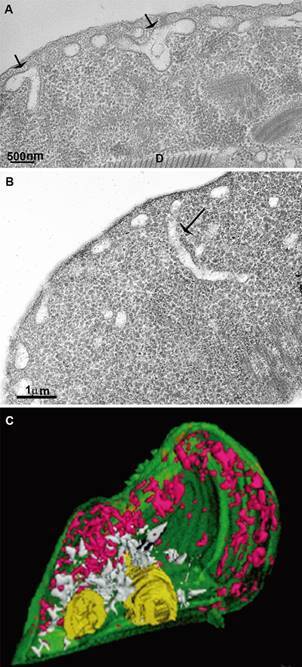
peripheral vesicles (PVs) of *Giardia intestinalis.* Some PVs are polymorphic (arrows in A), whereas others are tubular (arrow in B). The tridimensional reconstruction shows the polymorphism of the PVs (magenta). Endoplasmic reticulum (white), nuclear envelope (yellow). A-B: Benchimol (unpublished data); C: after.^20^

The endocytic system of *Giardia* has long been attributed to PVs, vesicular organelles of 150-200 nm size, placed just below the plasma membrane (Figs 1-3). They are found on the parasite’s dorsal side and in some ventral disc ventral regions, such as the bare zone.^17^ ^,^ ^18^ These vesicles are organelles that functionally correspond to *Giardia*’s endosomal-lysosomal system. This function was attributed to the typical characteristics of these organelles, such as the presence of hydrolase activity^19^ and its acidic nature demonstrated with acridine Orange observed by fluorescence microscopy.^20^ ^,^ ^21^ A membrane-bound cathepsin C activity was also detected in the acidic acid phosphatase-positive organelles.^22^ The endocytic capacity where PV can take small particles and macromolecules, such as ferritin particles, horseradish peroxidase, albumin, transferrin-coated colloidal gold particles, low density lipoprotein (LDL), fluorescent lipid analogues, and virus particles.^22^ ^,^ ^23^ Thus, the PVs function in fluid-phase endocytosis. A selective pathway sorting proteins from the plasma membrane to PVs has been demonstrated, such as a *Giardia* low-density lipoprotein receptor-related protein involved in selective lipoprotein endocytosis.^24^ ^,^ ^25^

Recently, Santos and co-workers,^26^ using distinct imaging techniques, reported that PVs are morphologically heterogeneous in size and shape (spherical, tubular, and polymorphic), claiming possible distinct functions and/or maturation states in this endocytic system. The authors proposed renaming PVs to peripheral endocytic compartments (PECs). The PVs heterogeneity has also been demonstrated previously.^20^

Data obtained by tomography, three-dimensional reconstruction, and ultrastructural cytochemistry using glucose-6-phosphatase staining (which labels ER) demonstrated that some PVs occur in tubular forms, and some are in continuity with the ER^18^ ^,^ ^20^ (Figs 3-4). Furthermore, the SRα protein, a recognition signal of ER-associated proteins, localises in some PVs.^27^ Interestingly, unlike classical eukaryotic cells, endosomal maturation in different compartments does not occur in *Giardia*.^28^

**Fig. 4:**
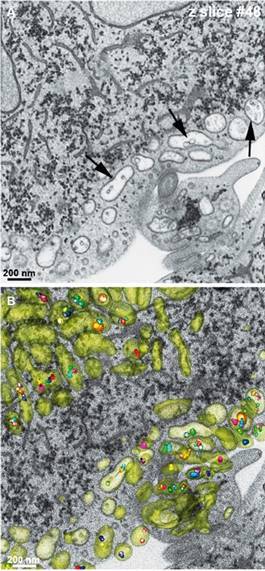
focused ion beam (FIB) and three-dimensional reconstruction of peripheral vesicles (PVs) (green) of *Giardia intestinalis* trophozoites. Notice nanovesicles inside PVs (arrows). The number of intra-vesicular bodies varied. After.^28^

Previous studies have shown that some vesicles use clathrin for endocytosis^29^ and the participation of a dynamin-related protein,^30^ which colocalises with clathrin in some PVs. In addition, Zumthor and co-workers^31^ reported that clathrin assemblies are small and focal clusters with no measurable turnover and exclusive cortical localisation distal to PVs. The authors suggested a possible novel function for clathrin in endocytosis.^31^

Microvesicles were described inside PVs using focused dual-beam microscopy, electron microscopy tomography, and 3D reconstruction^28^ (Fig. 4). Furthermore, the authors demonstrated that some PVs display MVBs, presenting a mean diameter of 50 nm and containing intraluminal vesicles (Fig. 4).

Until recently, all previous studies indicated that *Giardia* endocytoses are fluid-phase and receptor-mediated. Still, there has been no description concerning the interaction or ingestion of large materials or microorganisms. However, Benchimol^32^ recently reported that *Giardia* could interact with large particles and cells, such as yeasts, bacteria, and latex beads coated with ferritin and albumin proteins. In addition, although small markers, such as albumin, were found in the PVs, larger materials were seen inside distinct large vacuoles. Thus, it was demonstrated that *Giardia* interacts with large organic and inorganic materials and exhibits an amoeboid shape and membrane projections when in contact with microorganisms and large materials (Fig. 5). In addition, the opening exit of the ventral flagella seemed to be a preferential region for endocytosis,^32^ where clathrin is abundant and receptor-mediated endocytosis occurs.^18^

**Fig. 5:**
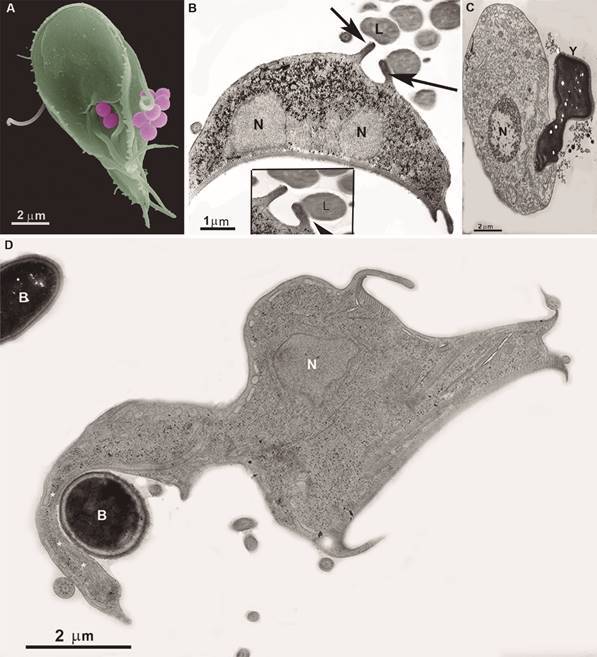
scanning (A) and transmission electron microscopy (B-D) of *Giardia intestinalis* in interaction with uncoated latex beads (A), latex beads coated with albumin (B), yeast (C), and bacteria (D). In (A), the latex beads are in the process of internalisation, just in the region of the ventral flagella exit. (B) plasma membrane expansions, like pseudopods, are in direction and contact with latex beads (L) (the inset shows it better). (C) A yeast (Y) is seen in the process of endocytosis by *G. intestinalis.* (D) A bacterium (B) is attached to a membrane expansion of *G. intestinalis.* Notice that the parasite has drastically changed its shape, presenting a long membrane extension, like a pseudopod, which presents filamentous structures actin-like (asterisks). N: nucleus. After.^32^

It is important to mention that this protozoan is grown in an axenic media in the laboratory, whereas *in vivo*, *Giardia* contacts a diversified microbiome. Thus, future research needs to answer questions about this parasite’s interactions with other microorganisms *in vivo*.

Cells produce and release several microvesicles and, among them, exosomes. Exosomes are of endosomal origin and are stored in MVBs as intraluminal vesicles (ILVs), released when the MVBs fuse with the plasma membrane. *Giardia* produces exosomes of the same size, shape, and protein and lipid composition as those described for other eukaryotic cells.

*Giardia* has a reduced ESCRT, and previous works suggested that *Giardia* exosome biogenesis is unique and occurs in the PVs.^33^ The authors also reported that *Giardia*, even lacking a classical endo-lysosomal system, can produce and release exosome-like vesicles.^33^

It is important to highlight the need to study the molecular and biochemical mechanisms involved in Giardia’s endocytic processes.

**Endocytosis in *Trichomonas***

The parasite protozoan *Trichomonas vaginalis* (Fig. 6) is the agent of trichomoniasis, an important sexually transmitted infection with about 156 million cases a year.^34^ Globally, trichomoniasis enormously impacts women as the most common non-viral sexually transmitted infection (STI). *T. vaginalis* causes vaginal discharge and has also been associated with adverse birth outcomes such as preterm birth, low birth weight, preterm rupture of membranes, and an increased risk of human immunodeficiency virus (HIV) acquisition. Despite advances in disease mechanisms, several questions still need to be answered. It is already established and well-known that trichomonads’ pathogenesis includes contact-dependent and independent mechanisms; the parasite ingests and digests cells and microorganisms^35^ (Fig. 6).

**Fig. 6:**
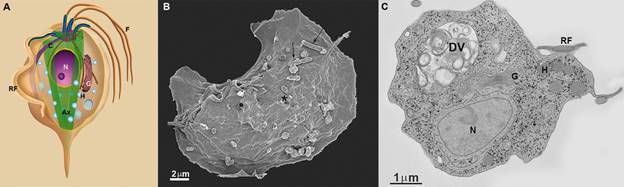
scheme (A), scanning electron microscopy (SEM) (B), and transmission electron microscopy (TEM) (C) of the endocytic activity of *Trichomonas vaginalis* when in contact with bacteria (arrows in A). The asterisk points to an endocytic pit. Notice the digestive vacuole (DV), where organic material is digested. Ax: axostyle; c: costa; G: Golgi; H: hydrogenosome; F: flagella; N: nucleus; RF: recurrent flagellum. Benchimol (unpublished data).

Endocytosis provides the parasite with important nutrients and contributes to its defensive responses to attacks from the immune system. In addition, previous studies demonstrated a relationship between phagocytic activity and the length of time the isolate is maintained in culture, *i.e.*, long-term and fresh cultures, where the virulence is lower depending on how often the culture is transferred.^36^

It has been reported that *T. vaginalis* can ingest latex beads as large as 4 µm in diameter,^37^ bacteria,^38^ ^,^ ^39^ and cells such as vaginal epithelial cells, leucocytes, and erythrocytes, among others^35^ ^,^ ^40^ ^,^ ^41^ (Figs 6-7) and yeast cells^36^ (Figs 8-9). Furthermore, *T. vaginalis* ingests various strains of *Neisseria gonorrhoeae* ^42^ and human viruses through an endocytic process.^43^

**Fig. 7:**
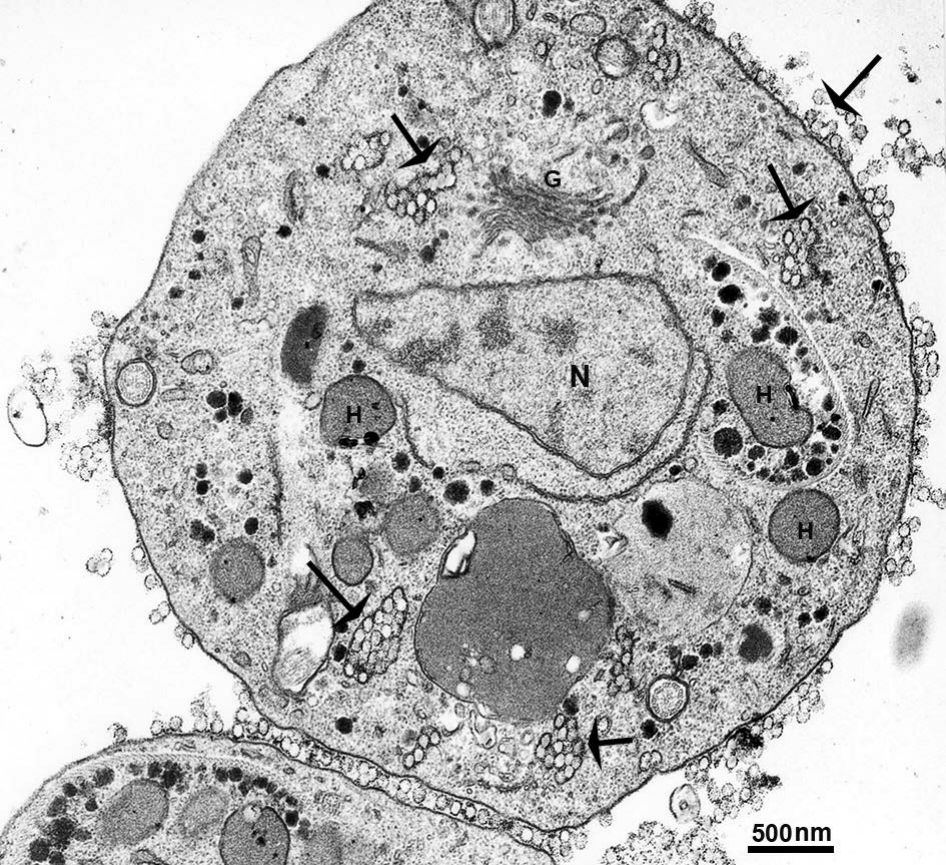
transmission electron microscopy (TEM) of the endocytic activity of *Trichomonas vaginalis* when in contact with ferritin-coated latex beads (arrows). Note that many látex beads are inside vacuoles and on the cell surface. Benchimol (unpublished data).

**Fig. 8:**
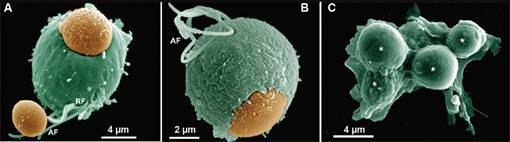
scanning electron microscopy (SEM) of the sinking process. *Trichomonas vaginalis* ingests the yeast *Saccharomyces cerevisiae* (orange) without any apparent participation of plasma membrane extensions (A-B). Notice that several yeasts (asterisks) can be ingested by the same cell (C). After.^36^

**Fig. 9:**
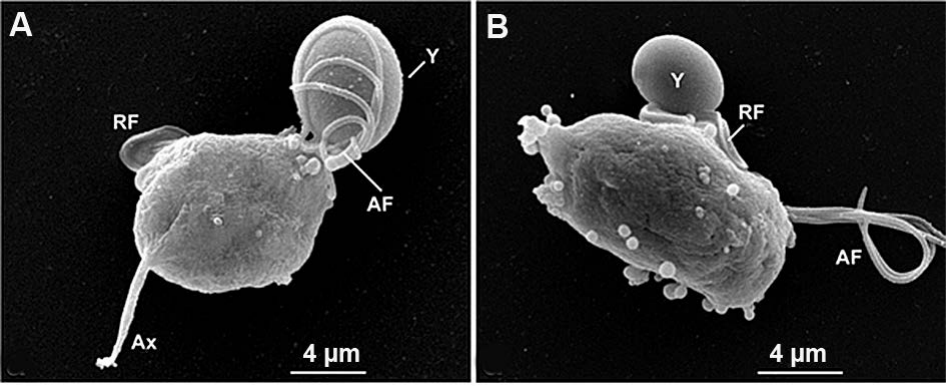
scanning electron microscopy (SEM) shows the attachment of *Saccharomyces cerevisiae* (Y) to the anterior (A) and recurrent flagellum (B) before the endocytic process. After.^36^

Viruses internalised by *T. vaginalis* were shown to be infectious for human cells and thus could contribute to their transmission to a new host.^43^ Patients infected with *T. vaginalis,* which contains human viruses, including HIV, accumulated in endosomal organelles,^43^ ^,^ ^44^ ^,^ ^45^ can transfer both parasites to a new host during sexual intercourse. The viruses are released upon parasite death or are secreted from infected *T. vaginalis* through the recycling route of the endocytic pathway.^46^ This study showed that viral NPs of HIV-1 enter trichomonads through focal condensations at the inner side of the plasma membrane, forming vesicles that look like clathrin-like coated pits. However, other clathrin-independent or receptor-dependent endocytosis pathways for NP internalisation are not excluded.

*Trichomonas vaginalis* exhibits two forms of phagocytosis: (1) classical phagocytosis, where pseudopodia are extended toward the target cell, and (2) an interesting process in which a ‘sinking’ process occurs without pseudopod formation.^36^ Using yeasts, the authors demonstrated a gradual ingestion of the whole yeast and several organisms in the same parasite. The process differs from classical phagocytosis since the yeast is gradually incorporated inside the parasite by sinking (Fig. 8).

The process of phagocytosis displays several phases: (1) the binding of material to be ingested to surface receptors of the phagocytic cell, which (2) triggers a series of events, cytoskeleton reorganisation with consequent pseudopod formation; (3) the pseudopods involve the material forming a phagosome, which fuses with the early and late endosomes, and next (4) the phagosome fuses with lysosomes, with the digestion of the internalised material.^47^ These four steps were also observed in the endocytic process by *T. vaginalis.*

Trichomonads can ingest small and large particles. The typical endocytic process involves the formation of small pits and vesicles, which fuse with lysosomes.^48^ Endocytic experiments using proteins, such as horseradish peroxidase and ferritin, coated gold particles with transferrin, lactoferrin, or LDL, showed the ability of trichomonas ingestion and digestion. It is important to mention that cultures recently isolated from patients exhibit intense endocytic activity and correlate with the grade of the parasite’s virulence.^35^

Studies carried out with trichomonads used different microscopy techniques to investigate the endocytic pathway in this protozoan. Among these techniques, we can cite the use of fluorescent molecules, small and large latex particles, gold-labelled macromolecules analysed by transmission electron microscopy of thin sections, and in freeze-fracture replicas, cytochemistry to label low pH compartments such as acid phosphatase and the DAMP technique^48^ to estimate the pH of intracellular compartments. In addition, the *T. vaginalis* genomic analysis revealed genes coding for proteins involved in the membrane trafficking machinery. However, genes that encode myosin were not detected.^49^ Several materials ingested by trichomonas follow a pathway like the early and late endosomes, ending in lysosomes, identified by their morphology, the accumulation of gold particles, and acid phosphatase cytochemistry.^48^ ^,^ ^50^ The *T. vaginalis* genome indicates this parasite has a complex degradome with more than 400 peptidases.^49^

It is important to highlight the need to study the molecular and biochemical mechanisms involved in Trichomonads’s endocytic processes.

It is well-known that *T. vaginalis* undergoes a radical morphological change when in contact with the host cell and during phagocytosis.^35^ ^,^ ^51^ In this case, the parasite flattens and becomes an amoeba-like cell. However, it is important to note that low-virulence strains did not show a complete amoeboid morphology during phagocytosis, whereas the highly virulent strains did.^36^

In addition to flagellate-amoeboid transition upon infection, actin also actively glides across host tissue.^52^ Several reports documented this transformation and the participation of cytoskeleton filaments such as actin-binding proteins, alpha-actinin, coronin, and fimbrin in the parasite periphery.^52^ ^,^ ^53^ ^,^ ^54^ ^,^ ^55^ ^,^ ^56^ In addition, many genes encoded cytoskeletal-associated proteins, such as kinesin and dynein, were predicted in the genome of *T. vaginalis.* It has also been shown that the endocytosis of yeast cells was blocked when trichomonads were treated with cytochalasin D, which inhibits actin polymerization.^36^

Recently, Zimmann and co-workers^57^ reported the phagolysosomal proteome of *T. vaginalis*, indicating a high complexity of their phagolysosomes, biogenesis, and role in the unconventional secretion of cysteine peptidases. In this work, the authors showed that *T. vaginalis* lysosomal proteome presents 462 proteins sorted into 21 classes. In addition to proteases, lipases, phosphatases, and glycosidases, they also identified a large set of proteins involved in vesicular trafficking (80) and turnover of actin cytoskeleton rearrangement (29), indicating a dynamic phagolysosomal compartment. Furthermore, the cysteine protease TvCP2, which has been demonstrated previously to be secreted, was inhibited by chloroquine, thus increasing the intralysosomal pH. This finding indicated that TvCP2 secretion occurs through lysosomes rather than the classical secretory pathway. The authors also reported that, unlike other parasitic protists, *T. vaginalis* might utilise glycosylation as a recognition marker for lysosomal hydrolases.

The presence of fibrilar actinin in the cytoplasm of *T. vaginalis* has been reported*,* although it is more concentrated in the cell periphery, just below the plasma membrane. The actin-based cytoskeleton mediates phagocytosis, which is blocked when drugs that affect actin are used, such as cytochalasins.^36^ In addition, parasites might contact epithelial cells through the flagella,^58^ and both anterior and recurrent flagella participated in the endocytosis of the yeast cells.^36^

*Trichomonas vaginalis* can ingest and digest sperm cells.^59^ This demonstration was relevant since this activity could contribute to fertility problems reported by infected people and cattle.

In contrast with higher eukaryotic cells, where endocytosis stops during mitosis, *T. vaginalis* keeps ingesting yeast cells during any phase of the mitotic process; thus, the phagocytic process occurs simultaneously during the parasite’s mitosis.^36^

It has been suggested that the participation of mannose receptors in yeast phagocytosis by *T. vaginalis*.^36^ The authors demonstrated that when *T. vaginalis* was incubated with sugars that compete for the mannose receptors, the phagocytosis of non-sensitised yeast cells was partially inhibited. Thus, the authors suggested that the non-specific recognition and phagocytosis of yeast cells by *T. vaginalis* is mediated by a mannose receptor on the parasite surface.^36^ In addition to D-mannose incubation with other competitor sugars, such as L-fucose, the phagocytosis was inhibited. In addition, the parasites may express other lectin-like receptors on the cell’s surface that would recognise different sugars in yeast cells.^36^ For example, chloroquine, a lysosomotropic drug, downregulated the expression of mannose receptors within intracellular compartments and interrupted the recycling process. Thus, in *T. vaginalis*, chloroquine inhibited phagocytosis in a dose-dependent manner.^36^

Previous studies demonstrated how *T. vaginalis* exerts its cytopathic effect.^41^ ^,^ ^50^ First, the parasites attack the host cells and induce plasma membrane damage and cell death, which occurs by mechanical stress on the microvilli of the host cells.^41^ Next, fragments of the necrotic cells are ingested by phagocytosis, where trichomonads avidly ingest large portions of epithelial cells, such as the nucleus and organelles, that are rapidly digested in lysosomes. However, living or intact cells were not ingested.^50^

*Trichomonas vaginalis* can evade complement-mediated lysis, although the parasite genome does not possess a DNA sequence with homology to human protectin (CD59). However, in experimental procedures where mouse erythrocytes were exposed to the parasite, Ibáñez-Escribano and co-workers^60^ reported the ability of *T. vaginalis* to acquire host CD59. As the capability of *T. vaginalis* to phagocyte red cells is well-known,^35^ the acquisition of complement protectin probably occurred from the host cell. Thus, acquiring CD59 from the host by trichomonads could be a novel and additional defence method for the parasite using phagocytic phenomena.

Some studies indicated that some isolates of *T. vaginalis* may provide an environmental reservoir for pathogenic organisms, such as mycoplasmas and virus-like particles (VLPs). In addition, some groups demonstrated the existence of isolate-to-isolate differences in the mycoplasmas’ ability to invade host cells and survive intracellularly and reported that *Mycoplasma hominis* could infect and multiply within trichomonads, establishing a probable symbiotic relationship.^61^ ^,^ ^62^ Furthermore, Vancini and Benchimol^62^ indicated that *M. hominis* enters *T. vaginalis* cells by endocytosis using the terminal polar tip as an anchor to the parasite plasma. In contrast, in other situations, digestion did not occur. It has been suggested that *T. vaginalis* may serve as a carrier for other microorganisms and, thus, would be a Trojan horse of bacteria and other pathogens.^63^ In other biological systems, studies show how the presence of an intracellular microorganism interferes with the ability of the host cell to inhibit or not the proliferation of an infective agent. Thus, it is an interesting research area that has yet to be addressed for the infection of anaerobic protists by viruses and bacteria.

Previous studies using yeasts in interaction with *T. vaginalis* demonstrated the participation of flagella in the first phase of the phagocytic event (Fig. 8). Furthermore, all flagella touch the yeast apparently to fix it and promote its ingestion,^36^ thus suggesting an important function in the phagocytic event.

A trichomonacidal effect was observed when *T. vaginalis* endocyte nanoparticles (NP) coated with chitosan.^64^ In this investigation, flow cytometry and electron microscopy were used in the analyses. NPs coated with chitosan were internalised as early as 10 min after incubation with the parasite and induced significant morphological changes in the cell, contrary to uncoated NPs. In addition, *T. vaginalis* exhibited numerous pits on the plasma membrane, leakage of intracellular components, and parasitic death. The authors showed that nanoparticles (NPs) and metronidazole (MTZ) did not show any antagonism or synergy, probably because the mechanisms of activity of NPs and MTZ are different. After their internalisation, Chito20 NPs were degraded inside the parasite, leading to toxic products and cell lysis. These findings led the authors to propose a combination of NP chitosan-coated with metronidazole, the drug usually chosen in trichomonad treatment.^65^ Thus, endocytosis can be useful in future drug treatments.

**Trogocytosis in *T. vaginalis***

Trogocytosis is considered a different way of phagocytosis, and it is characterised by ingesting a part of a cell by nibbling. Trogocytosis differentiates from phagocytosis since, in phagocytosis, a whole cell is ingested, whereas, in trogocytosis (Fig. 10), only pieces of a cell’s prey are incorporated (see an excellent review in^65^). *T. vaginalis* can ingest organic material in both ways.^49^ It has been demonstrated that *T. vaginalis* ingests necrotic cells, cell debris, and organelles such as the nucleus and microvillus.^41^ ^,^ ^50^ The authors also demonstrated that in *T. vaginalis,* phagocytosis might occur from any side of the cell. Cytochemistry for acid phosphatase indicated that all organic material was addressed to lysosomes for digestion.

**Fig. 10:**
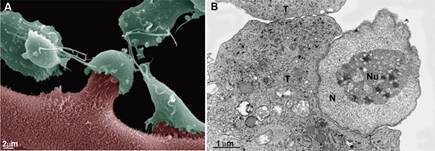
trogocytosis. (A) scanning electron microscopy (SEM) of a 1-h interaction between *Trichomonas vaginalis* (green) with a confluent monolayer of bovine oviduct epithelial cells (red). Notice that the parasite is pulling up the epithelial cells. (B) transmission electron microscopy (TEM) of trogocytosis: it is possible to note that large cell organelles, such as a nucleus, can be phagocytosed. After.^50^

In addition, a group (66) demonstrated trogocytosis using White cells on this parasite. The authors demonstrated that human neutrophils polymorphonuclear cells (PMNs) rapidly kill *T. vaginalis* in a dose-dependent, contact-dependent manner, forming a neutrophil extracellular trap (NET)-in an independent manner. The authors showed that in contrast to phagocytosis, PMN killing of *T. vaginalis* involved taking “bites” of *T. vaginalis* before parasite death using trogocytosis to kill the parasite. Both trogocytosis and parasite killing depend on the presence of PMN serine proteases and human serum factors.^66^ Recently, another group demonstrated that complement receptor 3 is required for human neutrophil-like cells’ maximum *in vitro* trogocytic killing of *T. vaginalis*.^67^

*Tritrichomonas foetus*

*Tritrichomonas foetus* (Fig. 11) is a parasite of cattle and other animals such as cats and pigs. It is an extracellular protozoan that colonises the preputial cavity and penis in males and the vagina and uterus in females. Infected bulls are generally asymptomatic, whereas infection in females eventually leads to abortion and infertility, with economic losses of up to 35%.^68^

**Fig. 11:**
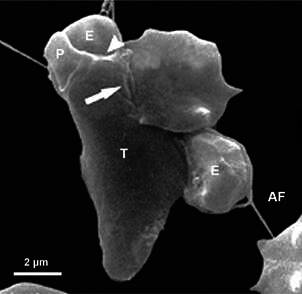
scheme (A) and scanning electron microscopy (SEM) of the interaction between *Tritrichomonas foetus* (T), K strain, and horse erythrocytes (E). The arrow points to a pseudopod formation. AF: anterior flagellum; N: nucleus; S: sigmoid filament; Ax: axostyle; H: hydro genome; Er: endoplasmic reticulum; UM: undulating membrane; L: lysosome; P: pelta; BB: basal body; PF: parabasal filament; F: cytoskeletal filaments. (A) Benchimol (unpublished data); (B) After.^71^

This parasite is of veterinary importance because it causes bovine trichomonosis, a major sexually transmitted disease in cattle. Bovine trichomonosis creates a serious global economic burden in areas where free-ranging herds are maintained using natural services for insemination.

*Tritrichomonas foetus* has a simple life cycle that consists of only a trophozoite form with three anterior flagela and one recurrent flagellum (Fig. 11). During unfavourable environmental conditions, the trophozoites, which are polar and flagellated, can change to a spherical shape and internalise their flagella. These rounded organisms are known as pseudocysts or endoflagellar forms.

Early studies of the endocytic activity of *T. foetus* demonstrated a lesser capacity to incorporate inert materials when compared with *T. vaginalis. T. foetus* can ingest polystyrene particles with a diameter of up to 1.0 μm.^37^ Using polystyrene spheres with a mean diameter of 4.4 μm, this group observed that when coated with several different ligands, the particles adhered to the surface but were not ingested. In addition, the authors showed that the parasite ingests large particles using a recognition system for fibronectin and laminin.^37^ They concluded that laminin and fibronectin might act as opsonising factors recognised by the parasite, increasing their ability to ingest components of the environment where they live.

*Tritrichomonas foetus* can also ingest diverse macromolecules.^69^ ^,^ ^70^ This group demonstrated that proteins were rapidly ingested within a few minutes through small vesicles and delivered to acid phosphatase-containing cytoplasmic vacuoles corresponding to lysosome-like organelles.^69^

In another study, the authors used scanning electron microscopy to show that human erythrocytes did not adhere to *T. foetus*. In contrast, horse erythrocytes adhered to the surface of the parasites (Fig. 11) and were phagocytosed for up to 90 min. The presence of pseudopods in *T. foetus* and the movement of the flagella suggested their importance for the uptake of nutrients from the surrounding medium. The authors also suggested that the axostyle could capture the erythrocyte.^71^

When *T. foetus* interacted with bovine and human sperm,^59^ cells obtained from uninfected bulls and men, respectively, over different periods, revealed a tropism, then proximity followed by a tight adhesion between these two different cells (Fig. 12). A decrease in spermatozoa motility was observed, as well as intense semen agglutination. The adhesion between trichomonads to the sperm cell occurs by the flagella or sperm head. Motile parasites were observed during the next 12 h, whereas sperm cells in contact with the parasites rapidly became immotile. The parasites could maintain the sperm cells attached to their cell surface, followed by phagocytosis and incorporation of the sperm cell within an intracellular vacuole (Fig. 12B). Afterward, the sperm cell was gradually digested in lysosomes. Many trichomonads were injured and/or died on contacting the spermatozoa, possibly due to necrosis. In this study, the authors demonstrated that both *T. foetus* and *T. vaginalis* interact with bovine sperm cells, provoking damage and death of these reproductive cells.^59^ Differences in the behaviour of both trichomonads showed that *T. vaginalis* was much more virulent than *T. foetus*. The possible role of trichomonads in reproductive failure was discussed. In another study, the authors demonstrated that *T. foetus* damages bovine oocytes *in vitro*.^72^

**Fig. 12:**
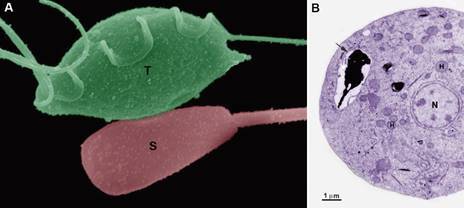
scanning electron microscopy (SEM) of a bovine sperm cell (S) in close contact with *Tritrichomonas foetus* after 30 min of interaction. (B) Transmission electron microscopy: after phagocytosis, remains of the sperm cell are seen inside an intracellular vacuole. Modified after.^59^

Concerning endocytosis kinetic, data from the literature indicate that some strains of trichomonads have an intense and fast endocytic activity throughout the protozoan surface. Typically, it takes a few minutes. After 2 min of incubation, gold-labelled transferrin was found in budding vesicles and tubule vesicular compartments of *T. foetus* (69). After 30 min at 37ºC, several compartments are full of the tracers. Latex particles were ingested by *T. foetus* as short as 3-min-incubation.^73^ Furthermore, previous studies dealing with NP ingestion by trichomonads showed that cationic NPs first adhered to the cell surface due to electrostatic interactions with the *T. foetus* membrane. NPs were seen after 3-min incubation.^73^

**Endocytosis in *Entamoeba***

*Entamoeba histolytica* (Fig. 13) is an enteric parasite capable of invading intestinal mucosa and spreading to other organs, mainly the liver. According to the World Health Organization (WHO), 500 million people could be infected with *Entamoeba* worldwide. *E. histolytica* can affect travellers who visit developing countries where amoebiasis is endemic. Amoebiasis causes 40,000-100,000 deaths annually and is the fourth leading cause of death due to a protozoan infection.^74^ ^,^ ^75^ It significantly affects morbidity and mortality in developing countries.^75^ The parasite presents two forms: the trophozoite (Fig. 13), the motile form, and the cyst, the resistance form. The trophozoite in the lumen of the large intestine, which multiplies and differentiates into a cyst, is the resistance form responsible for transmitting the infection. Trophozoites can invade the intestinal mucosa and spread to other organs.^74^

**Fig. 13:**
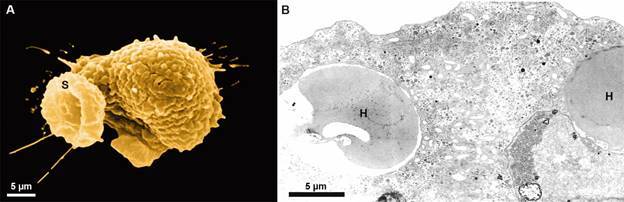
*Entamoeba histolytica* is seen by scanning electron microscopy (SEM) (A) and transmission electron microscopy (TEM) (B). Notice the stoma (S) formed during the phagocytic process (A) and the ingested red cells (H) in the vacuoles. Benchimol (unpublished data).

*Entamoeba histolytica* infection is multifactorial and depends on the interaction among the parasite, host, microbiota, or other pathogenic microorganisms. Much information has been obtained regarding the virulence factors, metabolism, and mechanisms of this parasite’s pathogenicity.^74^

*Entamoeba histolytica* initiates the infection in a cyst-resistant form, normally when one person ingests contaminated water or food. Excystation occurs in the terminal ileum, where motile and potentially invasive trophozoites are generated from the cysts. In this place, the parasite proliferates and adheres to the mucosal surface, invading the large intestine and causing diarrhoea and colitis.

Since the beginning of the 19th century, the ability of certain unicellular organisms to ingest cellular components or even whole cells as a nutritional source has been known. The article by Mast & Root^76^ describes ingesting bacteria and even part of a Paramecium by free-living amoebas (*Amoeba proteus*). This description characterises what we today consider to be one of the processes of endocytosis, phagocytosis, and trogocytosis (Fig. 14A). *Entamoeba histolytica*, in addition to these processes, carries out intense endocytosis of the pinocytosis type, evidenced by many vesicles of different sizes located in the cytoplasm of the trophozoite form and which can be visualised by light and electron microscopy^77^ ^,^ ^78^ (Fig. 14B). This protozoan is estimated to ingest fluids corresponding to about 15% of its volume in just 2 h.^78^ It is also possible to notice, especially in samples isolated directly from patients infected with pathogenic strains, that remnants of epithelial cells and erythrocytes can be seen within the cytoplasmic vacuoles, indicating their phagocytic nature.^79^ Experiments carried out using axenic cultures of *E. histolytica* showed, using transmission electron microscopy, that this protozoan incorporates native ferritin, cationised ferritin,^79^ colloidal gold particles coated with albumin, transferrin, lactoferrin, and LDL^80^ (Fig. 14C-F). Latex particles of variable diameters, bacteria,^81^ erythrocytes (Figs 13B, 15),^82^ ^,^ ^83^ and even epithelial cells and cells from the immune system^84^ are also ingested. These observations show the intense endocytic activity that takes place in this protozoan. Transferrin-binding proteins have been identified.^85^ Cytochemical analysis has shown that endocytic vesicles can be formed anywhere along the protozoan surface and that many are coated with clathrin^79^ (Fig. 14G). The vesicles fuse to form structures that can be recognised as early and late endosomes; later, their cargo is delivered to typical lysosomes (Fig. 14G). These vesicles are acidic, as evidenced by labelling with acridine orange, and accumulate external molecules taken by an endocytic process.^78^ ^,^ ^80^ The kinetics of acidification of the lysosomes varies according to the pathogenicity of the protozoan strain. Phagosomes from attenuated strains acidify rapidly within 2 min of formation.^86^ It has been shown that the endocytic activity in *E. histolytica* is a process that involves cell-signalling events and the participation of protein kinases and phosphatases.^87^ Indeed, treatment of the trophozoites with genistein, staurosporine, and Wortmannin, well-known inhibitors of protein kinases and PI-3 kinase, significantly inhibits the ingestion of erythrocytes.^82^

**Fig. 14:**
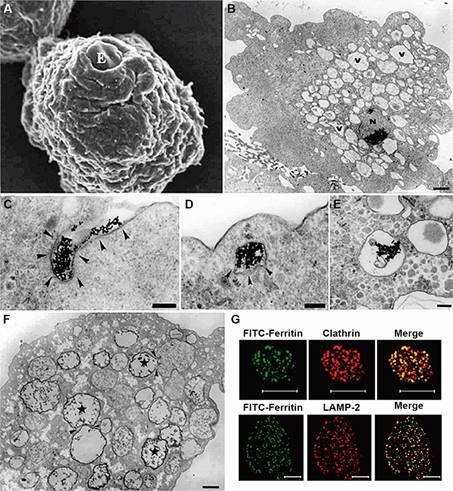
morphological characteristics of *Entamoeba histolytica* endocytic pathway. (A) Scanning electron micrograph (SEM) of an erythrocyte (E) phagocytosed by *E. histolytica*. After.^76^ (B) The overall appearance of the trophozoite forms is observed by transmission electron microscopy (TEM), revealing numerous vesicles, vacuoles, and glycogen particles within the cytoplasm. The nucleus and the presence of certain chromatoid bodies are also visible. Bars: 1.6 µm. (C-E) Endocytosis of lactoferrin coupled to gold particles shows the presence of the protein at tubular plasma membrane invaginations (C), inside vesicles in the cytosol (D), and in vacuoles at the central region of the cell, after 30 minutes of endocytosis (E). Bars: (C) 0,5 µm; (D) 0,17 µm; (E) 0,25 µm. (F) Fluid-phase endocytosis of HRP shows labelling in many vacuoles inside the cell (star), with some fusing with unlabeled ones (arrowheads). Bar: 1,65 µm. (B-F) After^74^ (G), FITC-Ferritin endocytosis is mediated by clathrin (upper panel), and ferritin is delivered to lysosomes after 30 min, marked by lysosomal-associated membrane protein 2 (LAMP-2) positive compartments (lower panel). Bars - upper panel: 24 µm; lower panel: 8 µm. After.^79^

Cytoskeletal components of the *E. histolytica* trophozoite are also involved in this endocytic activity. Prior parasite treatment with cytochalasins inhibits endocytic activity.^88^ ^,^ ^89^ Rapid actin polymerisation is observed upon contact of *E. histolytica* with target cells.^88^ Rho proteins are also involved in actin rearrangement; it was shown that antibodies recognising EhRhoA1 translocate from cytoplasmic vesicles to the protozoan plasma membrane.^90^ A gene coding for an unconventional myosin in *E. histolytica* has been described and characterised.^91^ ^,^ ^92^ Cell surface signalling and cytoskeleton involvement in the early steps of the phagocytic process have been well-established using several approaches.^93^ A calcium-binding protein (EhCaBP1) has been shown to participate in cellular processes involving actin filaments. The overexpression of this protein inhibits phagocytic activity but not fluid-phase pinocytosis.^94^ ^,^ ^95^ ^,^ ^96^

Components of the cytoskeleton, such as actin, play a relevant role in several stages of the endocytosis process that occur both in mammalian cells and in protists such as amoebae despite an evolutionary divergence of over a billion years. This similarity is reflected even in molecular details, as is the case with the participation of a family of proteins known as Rho, involved in controlling processes that require the participation of actin. In this context, it is important to remember that we find more similarities than differences when dealing with basic biological processes, even in different organisms. For example, the genomes of *Homo sapiens* and *Disctyostelium discoideum* point to 20 Rho GTPases in both.^97^ Of course, there are also differences. For example, mammals present different forms of actin, while *E. histolytica* has only one whose structure shows significant differences from the first (Review in ^98^).

Concerning the well-known phagocytic activity of *E. histolytica*, Bhararadwaj and colleagues^99^ identified the involvement of non-Dbl Rho guanine exchange factor (EhGEF) in regulating this activity. Using immunofluorescence microscopy, they show that EhGEF is localised in the phagocytic cup even during the progression of the cups and the closure of the phagosome, but not in the formed phagosome. Evidence indicated that EhGEF interacts with EhRho1 and is involved in the initiation of phagocytosis (Fig. 15A).

**Fig. 15:**
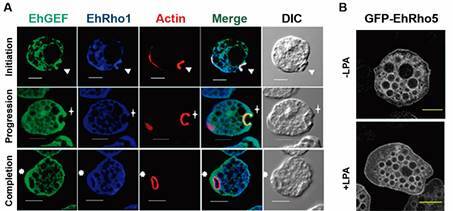
cytoskeleton participation in *Entamoeba histolytica* endocytosis. (A) Fluorescence images of EhGEF (green) with F-actin (red) and EhRho1 (blue) during different steps of erythrophagocytosis. Arrowheads indicate phagocytic cups, and asterisks mark actin enrichment in phagosomes. After.^93^ (B) Serum-starved GFP-EhRho5 trophozoites were stimulated in the presence and absence of 15 μM LPA. After.^100^. Bars: 10 µm.

EhRho5 is involved in constitutive and Lysophosphatidic acid (LPA)-stimulated macropinocytosis. Apte and colleagues used site-directed mutagenesis and RNAi to demonstrate this association. Upon LPA activation, EhRho5 undergoes translocation from the cytosol to the plasma membrane and endomembrane compartments. LPA signalling is mediated by a PI Kinase located upstream of EhRho5. Additionally, EhGEF2 serves as a guanine nucleotide exchange factor for EhRho5, and depleting it reduces the parasite’s macropinocytosis activity^100^ (Fig. 15B).

Recent studies have emphasised the importance of interaction between organelles in various cellular activities, including the endocytosis process, by establishing special contacts.^101^ ^,^ ^102^ Recently, it was reported the presence of a protein in the mitosome membrane of *E. histolytica*, designated as ETMP1 (*Entamoeba*-specific transmembrane mitosomal protein 1), which interacts with a protein member of the *Entamoeba histolytica* domain (EHD) superfamily, EHD1, involved in endocytic processes. Gene tagging allowed them to show that this protein is in the membrane of mitosomes but that it can also exist in other vesicles^103^ (Fig. 16).

**Fig. 16:**
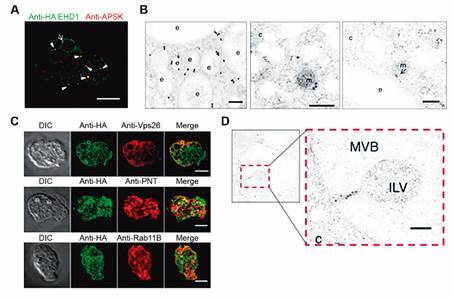
endosomes-mitosomes contact sites in *Entamoeba histolytica*. *E. histolytica* trophozoites expressing HA-tagged EHD1. (A) Immunofluorescence of fixed HA-EHD1-expressing cells double-stained with anti-HA (green) and anti-APSK (red). The arrow and arrowheads indicate proximity and colocalisation between anti-HA and anti-APSK signals, respectively. (B) Immunogold of HA-EHD1 trophozoites labeling anti-HA 5 nm gold and anti-APSK 15 nm gold. c: cytosol; e: endosome; m: mitosome. The arrow points to the structure where the membranes of the mitosome and endosome are in close contact. (C) Double labelling immunofluorescence of HA-EHD1 trophozoites with anti-HA and anti-endosomal markers antibodies. (D) Immunogold of HA-EHD1 trophozoites labelled with 15-nm gold-anti-HA. c: cytosol; MVB: multivesicular body; ILV: intraluminal vesicle. Bars: (A,C) 10 µm; (B,D) 200 nm. After.^103^

Lipid transport proteins (LTPs) play a relevant role in the distribution of lipids among the various organelles and lipid homeostasis.^101^ ^,^ ^104^ Recently, Das and colleagues^105^ showed that two StAR-related lipid transfer domain-containing LTPs, designated as EhLTP1 and EhLTP3, are involved in endocytic and exocytic processes. Examples include phagocytosis and trogocytosis. EhLTP1 is involved in pinocytosis and cysteine protease secretion processes.

The trogocytosis process starts via interaction with a Gal/GalNAc lectin on the parasite’s surface.^106^ Although its mechanism is not completely understood, it is known that it is an active process that requires the participation of the cytoskeleton composed mainly of actin, signalling via phosphatidylinositide 5 kinase (PI3K) and C2 domain-containing protein kinase (C2PK), and the AGC family kinase EhAGK1.^107^ It was also shown that inhibition of lysosome acidification inhibits both trogocytosis, phagocytosis, and cell killing.^108^

Small GTP-binding proteins are found in all cells, playing an important role as molecular switches involved in cell proliferation, cytoskeletal assembly, dynamics, and intracellular membrane trafficking. They constitute a superfamily that includes Ras, Rho/Rac, Rab, Sar, Arf, and Ran. Rab GTPases constitute the largest group. It was shown that *E. histolytica* presents 91 putative Rab genes. Twenty-two genes showed greater than 40% sequence identity to human proteins. Verma and colleagues recently reviewed all available information on Rab GTPase of *E. histolytica*.^109^ For instance, while mammalian cells have only one gene for Rab7, *E. histolytica* has nine Rab7 genes (reviews in^110^ ^,^ ^111^ ^,^ ^112^).

EhRab7A is localised in a non-acidic compartment that fuses with lysosomes,^113^ while EhRab7B is exclusively localised in acidic vacuoles containing lysosomal proteins such as amoebapore-A and cysteine proteinase.^114^ Rab11B, which is involved in the secretion of cysteine proteinase, was found in non-acidic vesicles, which may correspond to recycling endosomes.^115^ Rab5A, Rab7, and Rab11B were identified in isolated *E. histolytica* phagosomes.^114^ ^,^ ^116^ ^,^ ^117^

Perspectives

The data discussed in this review show that the endocytic process in pathogenic protozoa has been the subject of studies by several groups. Each parasite group developed diverse strategies for the early events of the endocytic process. Other protists, such as *Entamoeba* and *Giardia*, use a more classical endocytic process to form endocytic vesicles throughout most of the protozoan surface. The available data shows that for some protozoa, the studies are still basic, trying to describe, mainly using fluorescence and electron microscopy, the routes of the endocytic pathway. Certainly, these studies will benefit from using the recently developed expansion microscopy. In the case of *E. histolytica*, the studies are more in-depth, involving molecular analyses and knockout or overexpression of genes that regulate the endocytic process. This approach is expected to be extended to all protozoa in the coming years, especially with the broader use of CRISPR-Cas-9 technology. On the other hand, structural studies with the use of modern cryo-electron microscopy techniques will provide more precise structural information to understand the dynamics of the participation of several protein complexes involved in the various steps of the endocytic activity and whose structure can be analysed inside cryofixed cells, in a global context of the cell.

One important gap in the studies on anaerobic parasites is the lack of molecular mechanisms involved in endocytosis in *Giardia* and *Trichomonas*, which differs from endocytosis in *E. histolytica.* There are more studies at genomic and molecular levels in amoebas than in the other flagellates, and most studies describe morphology and phenomena but not mechanisms.
